# Platelet Function and Risk of Bleeding in Patients With Acute Coronary Syndrome Following Tirofiban Infusion

**DOI:** 10.3389/fphar.2019.01158

**Published:** 2019-10-09

**Authors:** Xiaoye Li, Shuning Zhang, Zi Wang, Qiuyi Ji, Qibing Wang, Xiaoyu Li, Qianzhou Lv

**Affiliations:** ^1^Department of Pharmacy, Zhongshan Hospital, Fudan University, Shanghai, China; ^2^Department of Cardiology, Zhongshan Hospital, Fudan University, Shanghai, China

**Keywords:** tirofiban, acute coronary syndrome, antiplatelet, bleeding risks, major adverse cardiovascular events

## Abstract

**Aim:** To assess platelet (PLT) function and bleeding risks in patients with acute coronary syndrome after tirofiban infusion.

**Methods:** Patients diagnosed with acute coronary syndrome from May 2016 to February 2018 in the Department of Cardiac Intensive Care Unit, Zhongshan Hospital, were enrolled. They were symmetrically allocated into two groups: tirofiban treatment group or control group (without tirofiban treatment). Blood samples were collected 24 h postoperation for the evaluation of antiplatelet effect of tirofiban. We applied thromboelastography to detect on-treatment PLT reactivity and conducted laboratory tests to assess the risk of bleeding following tirofiban treatment. After discharge, telephone follow-up and outpatient interview were carried out. The primary clinical endpoint was major adverse cardiovascular events, including cardiovascular death, cardiovascular mortality, myocardial infarction, and revascularization for the targeted vascular lesion.

**Results:** There were a total of 196 patients with acute coronary syndrome after screening with the inclusion criteria and the exclusion criteria. Ninety-eight patients were assigned to receive either tirofiban treatment or control treatment. Patients treated with tirofiban had more coronary lesions and stents implanted compared with the control group (*P* = 0.000). After tirofiban infusion, inhibition of platelet aggregation induced by thromboxane A_2_ and adenosine diphosphate was significantly higher compared to patients without tirofiban infusion (80.3% ± 19.6% vs. 72.6% ± 13.0%, *P* = 0.002; and 81.0% ± 19.8% vs. 75.4% ± 12.4%, *P* = 0.020, respectively). There was no significant difference in the reduction of hemoglobin (Hb), hematocrit (Hct), and PLT after administration of tirofiban, compared with baseline (*P* > 0.05). In addition, no significant differences were identified between the two groups with respect to Hb, Hct, and PLT after tirofiban infusion. However, C-reactive protein level, referred to as an inflammation marker, was significantly lowered after infusion tirofiban compared with the control group (11.9 ± 14.2 vs. 17.9 ± 21.2, *P* = 0.020). During the 1-year follow-up, the incidence rate of major adverse cardiovascular events remains indiscriminate between the two groups (*P* = 0.208). The assessments of cardiac biomarkers showed that tirofiban could decrease incidence of procedural myocardial infarction (odds ratio [OR] = 0.250, 95% confidence interval [CI] = 0.067–0.925, *P* = 0.027). At follow-up, the morbidity of left atrial dilation in tirofiban-treated patients, defined as enlargement of left atrial diameter >40mm, was lower compared to the control group (OR = 0.533, 95% CI = 0.301–0.945, *P* = 0.031).

**Conclusion:** Tirofiban infusion could decrease PLT activation in patients with acute coronary syndrome without increasing the risk of bleeding. As a concomitant medication, tirofiban shows no benefit in reducing the occurrence of major adverse cardiovascular events.

## Introduction

Acute coronary syndrome (ACS) has become the life-threatening disorder with high mortality and disability rate, which also causes significant economic burden annually (Turpie, 2006; Gao et al., 2018 ). The pathophysiology of ACS is in most cases due to the erosion or rupture of a plaque with consequent thrombotic obstruction of coronary artery (Seguchi and Kitakaze, 2005). Upon plaque rupture and erosion, platelet (PLT) activation and aggregation will lead to the coronary thrombosis. As PLTs play an important role in the progression of ACS, antiplatelet therapy could decrease the incidence and mortality of ACS (Amsterdam et al., 2014; Bittl et al., 2016).

Regular dual antiplatelet agents, aspirin and clopidogrel, could inhibit PLT activation and aggregation by different pathways on the PLT receptor of thromboxane and adenosine diphosphate (ADP) (Lamberts et al., 2014). Many current clinical trials indicated that the addition of tirofiban, a glycoprotein (GP) IIb/IIIa receptor antagonist, could effectively reduce ischemic events for patients with high risk of developing thrombosis (Januzzi et al., 2002; Servoss et al., 2004). Tirofiban is a nonpeptide drug with an l-tyrosine–modified structure widely used as a GPIIb/IIIa receptor antagonist. It inhibits the formation of PLT-specific integrin, which binds to protein ligands with adhesion properties, namely, fibrinogen, fibronectin, von Willebrand factor, and vitronectin (Choi et al., 2013). Tirofiban can provide a rapid, intensive PLT inhibition through reversible antagonist of fibrinogen binding to GPIIb/IIIa receptor, thereby blocking the final common pathway of PLT aggregation (Centurión, 2016).

Although several meta-analyses of randomized trials (Montalescot et al., 2007; De Luca et al., 2009) showed that periprocedural administration of GPIIb/IIIa inhibitor can improve the outcome of patients in terms of reinfarction and survival in relation to the patient risk profile, other clinical trials did not show any differences in mortality and reinfarction or major bleeding events in patients undergoing primary angioplasty. Current guidelines recommend that GPIIb/IIIa inhibitors should be taken into consideration in cases of no-reflow or a thrombotic complication (class IIa) (Roffi et al., 2015). There is currently paucity of large-scale studies comparing the clinical antiplatelet effect and safety of GPIIb/IIIa antagonists as well as studies investigating the effect of GPIIb/IIIa antagonists on the prognosis of myocardial infarction. This study evaluated the antiplatelet effect, risk of bleeding and major adverse cardiovascular events (MACEs) after intravenous tirofiban infusion.

## Methods

### Patient Population

This single-center, prospective cohort study recruited patients who were diagnosed with ACS according to the European Society of Cardiology (ESC) guidelines (Roffi et al., 2015) in the Department of Cardiac Intensive Care Unit, Zhongshan Hospital, Fudan University, between May 2016 and February 2018. All the patients presented with the symptoms of chest pain or discomfort, shortness of breath, dizziness, nausea, or sweating and were planned to undergo percutaneous coronary intervention (PCI). To be enrolled in this study, the inclusion criteria are as follows: 1) age ≥18 years, 2) no antiplatelet treatment history, 3) chest pain ≥10 min, 4) change in ST segment and T wave of electrocardiogram, 5) elevation of cardiac biomarkers, and 6) receiving loading dose of aspirin and clopidogrel before PCI operation. The exclusion criteria are as follows: 1) history of bleeding or hemorrhagic disease, 2) upstream use of antiplatelet drugs, 3) claiming coronary artery bypass surgery history, 4) PLT count <150,000/mm^3^, 5) prothrombin time >1.5 times control, and 6) hematocrit (Hct) < 30%. Clopidogrel at a loading dose (LD) of 300 mg was administrated before PCI, and a maintenance dose (MD) of 75 mg/d was administrated after PCI. Besides, all the enrolled patients were prescribed with the concomitant drugs including aspirin (LD/MD: 300/100 mg), low-molecular-weight heparin, renin–angiotensin–aldosterone system antagonists, statins, and β-blockers after admission to our hospital.

### Study Design

The eligible ACS patients were admitted to cardiac catheterization room for PCI operation, and stents were implanted on the occlusion coronary artery with stenosis >70%. Intravenous tirofiban was administrated according to the surgeon’s evaluation of thrombus burden and blood flow based on the level of stenosis in culprit vessels and multicoronary lesions. Patients were allocated 1:1 to the tirofiban group and control group.

In the tirofiban group, intravenous loading bolus 25 µg/kg of tirofiban was administrated within 5 min for the high-risk thrombosis patients during the operator progress, and postinfusion dose was 0.15 µg/kg/min for up to 18 h. Blood samples were collected for the evaluation of the antiplatelet effect of tirofiban 24 h after PCI operation.

Detailed information about each subject, including history of smoking or alcohol consumption, comorbidity disease, levels of hemoglobin (Hb) and Hct, PLT count, alanine aminotransferase, estimated glomerular filtration rate, and cardiac biomarkers as well as concomitant drugs in use, were collected through electronic medical records. We applied echocardiography to evaluate cardiac function including left atrial diameter and left ventricular ejection fraction (LVEF) during follow-up.

This study was conducted in compliance with the Declaration of Helsinki and Good Clinical Practice and approved by the Ethic Committee of Zhongshan Hospital. The written informed consent was signed by every participant.

### Blood Sample Collection

Approximately 4 ml of whole venous blood samples were collected into Vacutainers containing anticoagulant ethylene diaminetetraacetic acid (EDTA) from all patients upon recruitment to detect the on-treatment PLT reactivity with thromboelastography (TEG; Haemoscope Corp, Niles, IL, USA).

### Antiplatelet Measurements

Inhibition of PLT aggregation was analyzed according to manufacturer’s instructions. We added the following agonists as PLT stimulators: ADP (20 μmol/L) and thromboxaneA_2_ (TXA_2_; 1 mmol/L). The largest change in the value of the amplitude of coagulation intensity was defined as the maximum clot strength (MA). The MA was classified into the thrombin-induced maximum coagulation strength (MA_thrombin_), the activator-induced maximum coagulation strength (MA), and the fibrin-induced maximum coagulation strength (MA_Fibrin_), according to different types of activators (ADP and TXA_2_) added to the blood sample. The IPA was calculated by the formula: inhibition of PLT aggregation (IPA) (%) = (MA − MA_Fibrin_)/(MA_Thrombin_ − MA_Fibrin_) (Lu et al., 2013).

### Clinical Outcomes

Follow-ups were made *via* telephone and outpatient interview after discharge. The primary clinical endpoint was MACE, including cardiovascular death, cardiovascular mortality, myocardial infarction, and revascularization for the targeted vascular lesion (Park et al., 2011). Thrombolysis in Myocardial Infarction (TIMI) bleeding was defined as a bleeding event associated with decrease in Hb or Hct values (Hochholzer et al., 2011).

### Study Endpoints

The primary endpoint definition was comparison of IPA stimulated by ADP and TXA_2_ 24 h after PCI operation. According to previous studies, IPA_(ADP)_ < 30% and IPA_(TXA2)_ < 50% were defined as high on-treatment PLT reactivity (HPR) (Lv et al., 2016). The Hb, Hct, and PLT levels after PCI as compared with baseline in each group were evaluated between two groups. The second endpoint was the occurrence of target vessel revascularization or stent thrombosis at 12 months and bleeding complications according to TIMI criteria.

### Sample Size Calculation

Our study was designed on the basis of the noninferiority principle (the without-tirofiban group was considered noninferior to the tirofiban group) (Marian et al., 2017). For the sample size calculation, the primary endpoint (IPA after tirofiban infusion) was set at 95%. Based on a power of 85% and an α level of 0.05, at least 89 patients were required in each group to reach statistical significance. Considering a 10% dropout rate, 98 patients were enrolled in each group, and they all met the inclusion criteria and exclusion criteria.

### Statistical Analysis

The numerical data are presented as mean ± SD. Categorical variables are expressed with frequencies ± percentage. Independent *t* test was used in parametric data, and Mann–Whitney *U* test was used in nonparametric data. The baseline and angiography procedural characteristics were compared between two groups. The cutoff value of TXA_2_ and ADP high sensitive test from TEG was considered to be 50% and 30%, respectively (Lu et al., 2013). Results of antiplatelet effect induced by TXA_2_ and ADP were compared by *t* test.

The comparison of Hb and Hct levels changes, C-reactive protein, and bleeding complications were analyzed by Student *t* test. We analyzed MACE using multiple statistical models. Variables including occurrence of new abnormal Q-waves, cardiac biomarkers, LVEF, and left atrial dilation were analyzed. Hazard ratios (HRs) with two-sided 95% confidence intervals (CIs) were calculated for the risk factors of MACE. Results are presented as HRs along with 95% CI. Survival curves were constructed using Kaplan–Meier estimates. A two-sided *P* value was used to determine significance (threshold, *P* < 0.05). Statistical analysis was performed using SPSS (IBM SPSS Statistics 22.0) and Prism 5 (GrandPad Software). A *P* value of 0.05 was considered to be the threshold for statistical significance.

## Results

### Patient Characteristics

Our enrollment began in May 2016 and ended in February 2018. A total of 196 ACS patients who met both the inclusion criteria and the exclusion criteria were enrolled and symmetrically divided to two groups: 98 patients were assigned to receive tirofiban and 98 were in the control group. All the clinical data were presented in **Supplementary Table 1**. Comparison of baseline characteristics is shown in **Table 1**. There was no significant difference between the two groups with respect to age, gender, body mass index, cardiovascular risk factors, cardiac biomarkers, concomitant drug, and other characteristics.

**Table 1:** Demographic and baseline characteristics comparison.

Characteristics	Tirofiban (n = 98)	Control (n = 98)	*P*
Age (y)	64.0 ± 11.4	64.8 ± 11.0	0.598
Gender (male, %)	84.7%	78.5%	0.268
BMI (%)	24.4 ± 3.6	24.9 ± 3.6	0.310
HTN (%)	57.1%	70.4%	0.053
DM (%)	29.6%	39.8%	0.175
Hyperlipidemia (%)	10.2%	7.1%	0.446
Smoking (%)	51.0%	47.9%	0.668
Alcohol (%)	20.4%	19.4%	0.718
ALT (IU/L)	47.6 ± 42.1	41.7 ± 52.8	0.398
eGFR (ml/min·1.73 m^2^)	82.6 ± 19.2	86.2 ± 21.4	0.219
cTnT (μg/L)	12.5 ± 6.1	12.4 ± 3.2	0.249
CK-MB (U/L)	148.3 ± 139.5	145.1 ± 95.1	0.849
Concomitant drug			
Aspirin (%)	92.8%	95.9%	0.551
Clopidogrel (%)	70.4%	81.6%	0.066
Ticagrelor (%)	28.5%	18.3%	0.092
Statins (%)	100%	100%	1.000
ACEI/ARB (%)	88.7%	79.6%	0.078
β-Blocker (%)	93.8%	89.8%	0.408
PPI (%)	87.7%	90.8%	0.488

BMI, body mass index; HTN, hypertension; DM, diabetes mellitus; ALT, alanine aminotransferase; eGFR, estimated glomerular filtration rate; cTnT, cardiac troponin T; CK-MB, creatine kinase–MB; ACEI, angiotensin-converting enzyme inhibitor; ARB, angiotensin receptor blockers; PPI, proton-pump inhibitors.

### Angiography and Procedural Characteristics

All enrolled patients underwent PCI operation, and stents were implanted on culprit lesions. There was no significant difference between the two groups (tirofiban group and control group) with respect to coronary occlusion, unfractionated heparin dose, number of stents, total stent length, lesion vessels, cardiac biomarker levels, days of hospitalization, and MACE ratio. Patients who received tirofiban were at higher risk of left anterior descending culprit lesions (59.2% vs. 30.6%, *P* = 0.000; **Table 2**). In addition, ACS patients receiving tirofiban treatment had more coronary lesions and stent implantations compared with the control group (*P* = 0.000; **Table 2**).

**Table 2:** Angiography characteristics comparison between tirofiban and control groups.

	Tirofiban (N = 98)	Control (N = 98)	*P*
LAD (%)	59.2%	30.6%	0.000*
LCX (%)	22.4%	26.5%	0.616
RCA (%)	40.8%	32.6%	0.236
LM (%)	8.1%	6.1%	0.579
No. of lesion coronary (n)	2.42 ± 1.0	2.1 ± 0.8	0.000*
UFH (U)	7,348.2 ± 1,936.2	7,187.8 ± 2,752.0	0.637
No. of stents (n)	1.78 ± 0.84	1.15 ± 0.60	0.000*
Stents length (mm)	47.7 ± 23.5	41.6 ± 27.8	0.102
cTnT (μg/L)	2.34 ± 2.58	1.84 ± 2.32	0.150
CK-MB (IU/L)	29.7 ± 20.1	36.6 ± 32.9	0.077
Days (n)	8.7 ± 4.1	9.5 ± 4.9	0.202
MACE (%)	11.2%	17.3%	0.221

LAD, left anterior descending; LCX, left circumflex artery; RCA, right coronary artery; LM, left main coronary artery; UFH, unfractionated heparin; cTnT, cardiac troponin T; CK-MB, creatine kinase–MB; CRP, C-reaction protein; MACE, major adverse cardiovascular events.

*P < 0.05.

### Antiplatelet Assessment by TEG

**Figure 1:**
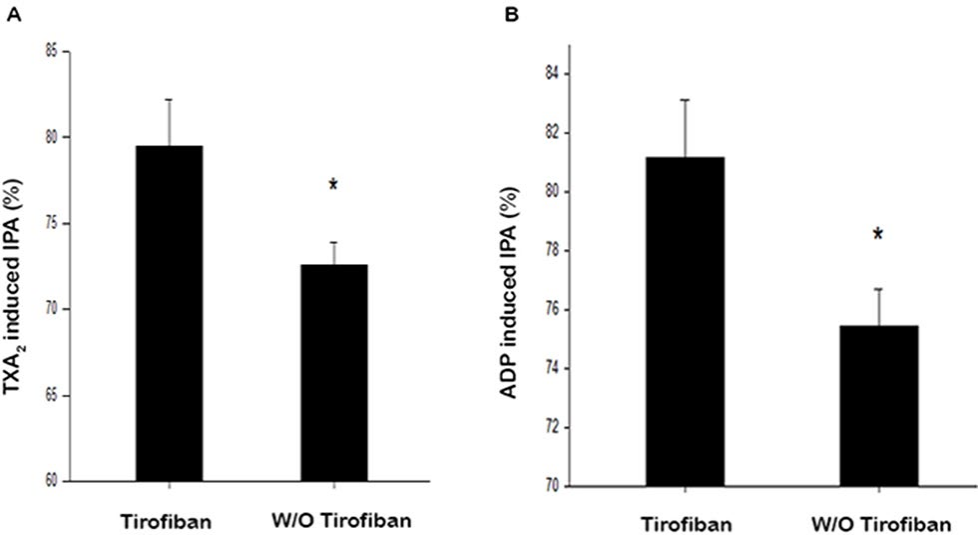
Antiplatelet effect of IPA with addition of TXA_2_ **(A)** and ADP. **(B)** TXA_2_, 1 mmol/L; ADP, 20 μmol/L; IPA, inhibition of platelet aggregation; *P < 0.05.

The percentage of HPR stimulated with TXA_2_ 1 mmol/L were 3.1% and 4.1% for the tirofiban and control groups, respectively (*P* = 0.997). As shown in **Figure 1A**, after tirofiban infusion, IPA induced by TXA_2_ was significantly higher compared to that in the control group (80.3% ± 19.6% vs. 72.6% ± 13.0%, *P* = 0.002). Meanwhile, the percentage of HPR stimulated with ADP 20 μmol/L were 1.0% and 1.0% for the tirofiban group and control group, respectively (*P* = 1.000). As shown in **Figure 1B**, ADP-induced IPA was significantly higher in the tirofiban group than that in the control group 24 h after PCI operation (81.0% ± 19.8% vs. 75.4% ± 12.4%, *P* = 0.020).

### Bleeding Assessment

**Figure 2:**
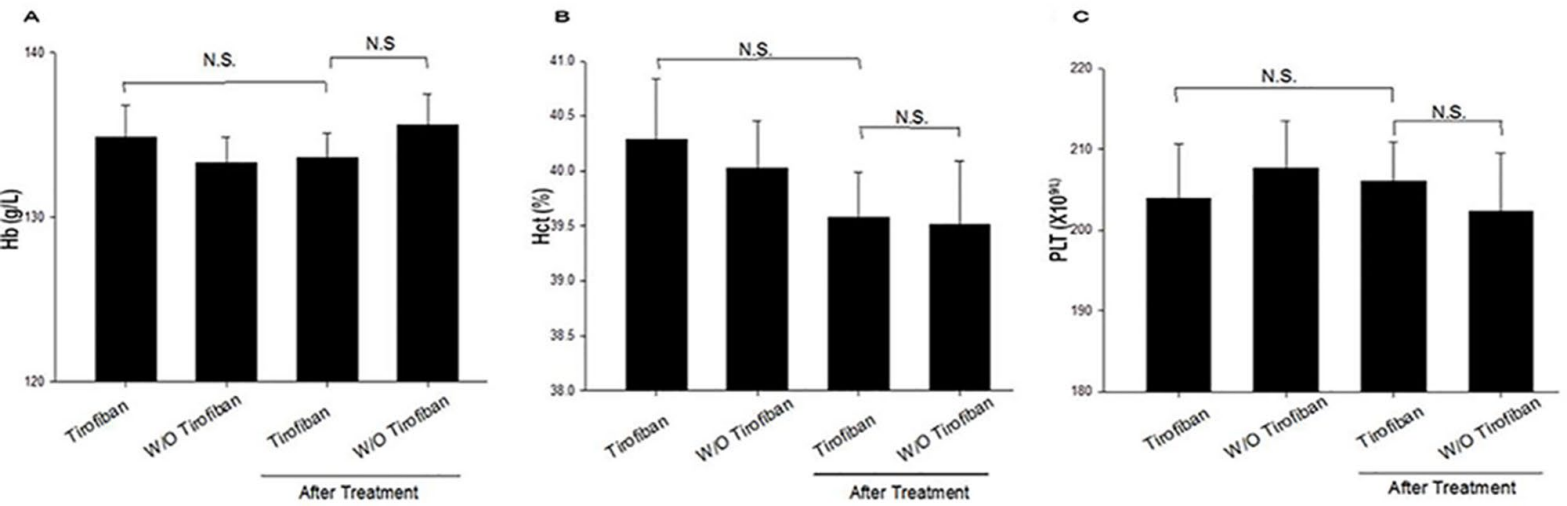
Laboratory characteristic comparison between baseline and treatment for the two groups **(A–C)**. Hb, hemoglobin; Hct, hematocrit; PLT, platelet; N.S., no significance.

As shown in **Figure 2**, the baseline values of Hb, Hct, and PLT were not significantly different between the groups (*P* > 0.05). After administration of tirofiban, decreases in Hb, Hct, and PLT were not significantly different compared with baseline (*P* > 0.05). There was no significant difference between the two groups with respect to the levels of Hb, Hct, and PLT 24 h after PCI.

### Inflammation Assessment

**Figure 3:**
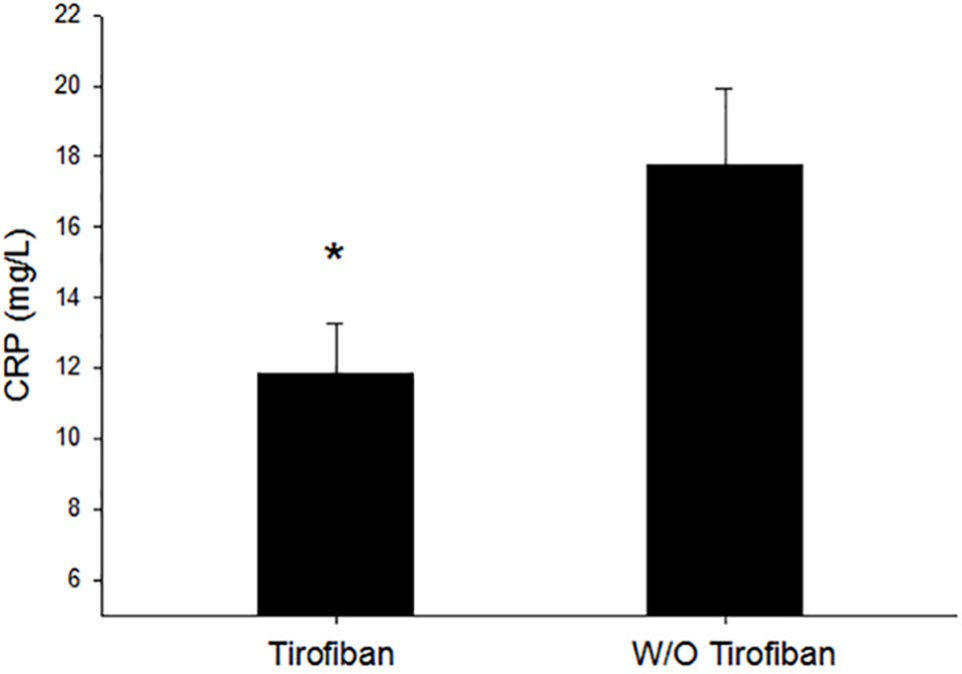
Inflammation comparison between the tirofiban group and control group. CRP, C-reactive protein; *P < 0.05.

As shown in **Figure 3**, inflammation biomarker and C-reactive protein values were significantly lower in the tirofiban group than that in the control group 24 h after PCI operation (11.9 ± 14.2 vs. 17.9 ± 21.2, *P* = 0.020).

### The Occurrence of MACE During the 1-Year Follow-Up

**Figure 4:**
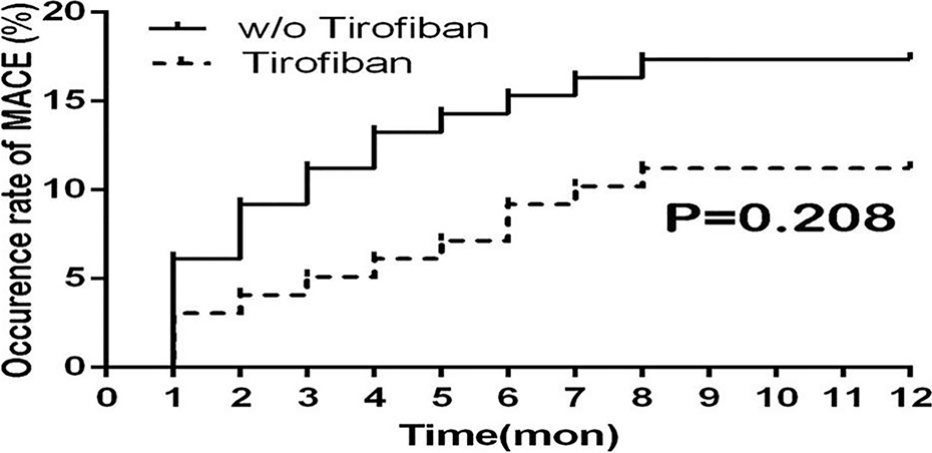
Survival curve of MACE ratio for 1-year follow-up between groups. MACE, major adverse cardiovascular events.

During the 1-year follow-up, the primary rates of MACE occurrence including the rehospitalization for the revascularization between the tirofiban group and control group were 11.2% and 17.3%, respectively. There was no significant difference after tirofiban administration in terms of MACE occurrence ratio compared with the control group (*P* = 0.208) As shown in **Figure 4**.

### Efficacy and Safety Evaluation

The assessments of cardiac biomarkers showed that tirofiban could decrease the incidence of procedural myocardial infarction [odds ratio (OR) = 0.250, 95% CI = 0.067–0.925, *P* = 0.027] (**Table 3**). Also for the follow-up, occurrence ratio of left atrial dilation defined as enlargement of left atrial diameter >40 mm was lower in the tirofiban group than in the control group (OR = 0.533, 95% CI = 0.301–0.945, *P* = 0.031) (**Table 3**).

**Table 3:** Clinical outcomes in patients between the tirofiban and control groups.

	Tirofiban, %	Control, %	OR	95% CI	*P*
Restenosis	2	6.1	0.319	0.063–1.623	0.279
Bleeding	10.2	7.1	1.477	0.538–4.053	0.613
3–5 Times normal	3.1	11.2	0.250	0.067–0.925	0.027*
5 Upper times normal	3.1	5.1	0.587	0.136–2.528	0.721
Abnormal Q wave	13.3	12.2	1.096	0.473–2.539	1
Left atrial (> 40 mm)	35.7	51	0.533	0.301–0.945	0.031*
LVEF (< 50%)	30.6	24.5	1.36	0.725–2.553	0.337

OR, odds ratio; CI, confidence interval; LVEF, left ventricular ejection fraction

*P < 0.05.

## Discussion

A large body of evidence is now available about the benefits of tirofiban application, in terms of the prevention of stent thrombosis and the reduction of MACE, especially among ACS patients undergoing PCI operation (Januzzi et al., 2002). Although the GPIIb/IIIa antagonists were developed to prevent PLT aggregation, increasing evidence supports the concept that GPIIb/IIIa antagonists can exert further biological effects and that PLTs are not the only targeted cells. Such effects may be, in part, dependent on integrin–transmembrane receptor triggering. In particular, the integrin-mediated induction of vascular endothelial growth factor (VEGF) stimulated by the arginine–glycine–aspartic acid (RGD) mimetic tirofiban appeared to be of considerable interest (Giordano et al., 2016).

Our study found that administration with IIb/IIIa blocker antagonist, tirofiban, could provide a stronger effect on PLT inhibition induced by TXA_2_ and ADP compared with treatment not using a GP inhibitor (GPI). In addition, the risk of bleeding was not aggravayed by tirofiban. Moreover, as shown in previous overall clinical trials (Januzzi et al., 2002; Servoss et al., 2004), ACS patients treated with tirofiban had a lower rate of MACE. Platelet GPIIb/IIIa receptor blocker can inhibit the formation of thromboembolism by preventing fibrinogen binding to PLT IIb/IIIa receptors, which is the final pathway for PLT aggregation (Starnes et al., 2011). In our study, tirofiban infusion had benefit on patients who were prone to develop restenosis after PCI operation. One registry reported that ACS patients treated with GPI had longer stents with larger diameters and more complexed lesions (Safley et al., 2015). A meta-analysis showed that mortality could be reduced by 25% for patients undergoing PCI who were confined at the highest risk (Sethi et al., 2013). Considering the high thrombosis burden mediated with PLT aggregation, current guidelines (Roffi et al., 2015; Bittl et al., 2016) recommend tirofiban infusion for high-risk ACS patients after PCI operation. However, most clinical trials mainly focused on the occurrence of adverse cardiovascular events. On top of this, our study also showed the effect of tirofiban on the inhibition of PLT aggregation induced by TXA_2_ and ADP. Our result demonstrated that administration of tirofiban induced a robust inhibition of PLT aggregation induced by TXA_2_. Various stimulations, including TXA_2_ and ADP, mediate PLT activity and are upstream pathways that can be modulated by specific antiplatelet therapy. Tirofiban, acting on final downstream pathway of PLT aggregation, can regulate PLT activation induced by TXA_2_. Patients medicated with tirofiban achieved statistically higher ADP-induced IPA. This phenomenon (Valgimigli et al., 2012) might be explained by the fact that tirofiban provides complementary antiplatelet effect through GPIIb/IIIa receptor affecting both the magnitude and the consistency of ADP-induced IPA. One randomized clinical trial (Holmes et al., 2015) revealed that after 24-h tirofiban infusion, ADP-induced PLT aggregation decreased from 95.5% to 28%. Another clinical trial (Marian et al., 2017) reported that ADP-induced PLT aggregation dropped from 99.8% to 59.4% in the high-risk non–ST-segment ACS patients 24 h after administration with GPI. Our results did not reflect such a significant drop compared with previously reported result (Marian et al., 2017), possibly because the P2Y12 receptor antagonist used in our study was clopidogrel, whereas in the previous study, novel oral antiplatelet drugs, such as prasugrel and ticagrelor, were used.

In this study, tirofiban inhibited the release of inflammatory cytokines such as CRP. One possible explanation might be that GPI can improve the function of vascular endothelium and increase the level of circulating endothelial progenitor cells mediated by inhibition of PLT aggregation (Jeong et al., 2017). Our result demonstrated that tirofiban infusion had potent and sustained suppressive effects on CRP levels following PCI. The main mechanism might be that elevated CRP levels had been correlated with vascular injury and PLT aggregation. The inflammatory sequence that follows vessel injury involved PLTs as well as leukocytes and is reflected in the temporal sequence of elevated serologic markers, including sCD40L, IL-6, and CRP (Kereiakes, 2006). The CRP levels might be directly correlated with the incidence of adverse ischemic outcomes in patients with ACS and could be a powerful predictor for clinical benefit associated with tirofiban therapy.

Besides the antithrombosis effect, the safety profile such as bleeding complications of tirofiban needs to be taken into account. Our results showed that no significant difference was found between groups with respect to laboratory biomarkers such as Hb, Hct, and PLT (*P* > 0.05). One meta-analysis reported (Winchester et al., 2012) that the use of tirofiban was not associated with an increase of major bleeding risk for ACS patients. Tirofiban has been shown to reduce MACEs for high-risk ACS patients when administrated in addition to aspirin and clopidogrel (Servoss et al., 2004). Previous study indicated that endothelial cells cultured with tirofiban showed increased proliferation. The growth-stimulating effect of tirofiban was mediated by VEGF production (Giordano et al., 2014). This cytokine was also responsible for increased endothelial cell migration, thus explaining the effect of the increased wound healing capability of endothelial cells stimulated with tirofiban. The stimulation of VEGF production was obtained at a dose of 0.25 μg/ml tirofiban (Giordano et al., 2016).

Tirofiban could provide a rapid onset of antiplatelet aggregation, which is a crucial benefit for a better clinical outcome of myocardial ischemia reperfusion. Moreover, it could provide 80% inhibition of PLT aggregation (Authors/Task Force Members et al., 2011; Wenger, 2012 ). Our results showed tirofiban could decrease the incidence of procedural myocardial infarction (OR = 0.250, 95% CI = 0.067–0.925, *P* = 0.027). Left atrial size enlargement was a predictor of mortality for both cardiovascular issues and all-cause mortality (Patel et al., 2009). We demonstrated that tirofiban decreased the rate of left atrial enlargement, which might be attributed to the reduced ratio of myocardial infarction.

The results of our study showed that administration of tirofiban, when infused immediately after PCI operation, could decrease the rate of reinfarction and mortality during the following 1 year. But no significant difference was found between the two groups (*P* = 0.208). It might be explained that the novel P2Y_12_ receptor blockers ticagrelor that provided faster, greater, and more consistent inhibition of PLT aggregation can obviate use of tirofiban for high-risk ACS patients (Wallentin et al., 2009). One clinical trial (ten Berg et al., 2010) reported that there was no significant difference in the decrease of mortality (3.7 vs. 5.8%, *P* = 0.08) after administration with tirofiban during 1-year follow-up. Our findings were consistent with previous reports.

## Conclusion

Our study showed that tirofiban infusion contributes to the inhibition of PLT aggregation in ACS patients 24 h after PCI operation, without elevating the risk of bleeding and concomitant administration of tirofiban with limited effect on decreasing the incidence of MACE in 1-year follow-up.

## Study Limitations

Our study had some limitations: First, it is a prospective single-center cohort study with a small sample size, which reduces the significance of our study. Second, the enrolled patients were not randomized to assigned groups. In the future, large prospective, randomized controlled trials are needed to evaluate clinical effect and adverse drug reaction of tirofiban administration. Third, patients with culprit vessel stenosis and multi–coronary lesions tended to receive medication with tirofiban. Fourth, long-term follow-up should be taken into consideration in future study. Fifth, we obtained some MACE information *via* telephone that could only roughly reflect the recovery of function.

## Data Availability Statement

The raw data supporting the conclusions of this manuscript will be made available by the authors, without undue reservation, to any qualified researcher.

## Ethics Statement

This study was carried out in accordance with the recommendations of “Comparison of antiplatelet regimens post percutaneous coronary intervention operation for acute coronary syndrome patients, approved no. B2016-002” with written informed consent from all subjects. All subjects gave written informed consent in accordance with the Declaration of Helsinki. The protocol was approved by the ethic committee of Zhongshan Hospital, Fudan University.

## Author Contributions

XyeL, SZ, and QL conceived the study and wrote the paper. ZW and QJ performed the experiments and contributed to the manuscript. QW and SZ enrolled the patients and collect information. XyuL contributed to the data statistical analysis.

## Funding

This study was supported by the Project of Shanghai Municipal health planning commission (NO.2016ZB0301).

## Conflict of Interest

The authors declare that the research was conducted in the absence of any commercial or financial relationships that could be construed as a potential conflict of interest.
